# Factors influencing the implementation of a pilot smoking cessation intervention among migrant workers in Chinese factories: a qualitative study

**DOI:** 10.1186/s12889-019-7223-1

**Published:** 2019-07-03

**Authors:** Guanyang Zou, Xiaolin Wei, Simin Deng, Jia Yin, Li Ling

**Affiliations:** 1 School of Economics and Management, Guangzhou University of Chinese Medicine, Guangzhou, 510000 Guangdong China; 2 Centre for Migrant Health Policy, Sun Yat-sen University, Guangzhou, 510000 Guangdong China; 3 Division of Clinical Epidemiology &Institute of Health Policy, Management and Evaluation, Dalla Lana School of Public Health, University of Toronto, Toronto, Ontario M2J 4A6 Canada; 4 Kangning Hospital, Shenzhen, 518000 Guangdong China; 5 School of Health Policy and Management, Shandong University, Jinan, 250000 Shandong China; 6 Department of Medical Statistics, School of Public Health, Sun Yat-sen University, Guangzhou, 510000 Guangdong China

**Keywords:** Smoking cessation, Intervention, Internal migrants, Factories, China

## Abstract

**Background:**

Tobacco control intervention with Chinese internal migrants, especially those working in factories has rarely been investigated. This study aims to identify aids and barriers to implementing a comprehensive pilot intervention aimed at reducing smoking among migrant workers working in factories in China.

**Method:**

Twenty in-depth interviews were conducted 3 months into the intervention, with managers, migrant workers and team leaders in two factories, where the pilot intervention was implemented, in Zhongshan city in Guangdong, a southern Chinese province. Data analysis was based on the thematic approach.

**Results:**

This study identifies the societal, individual and programmatic factors that could influence the implementation of a pilot smoking cessation intervention among migrant workers in the two Chinese factories. At the societal level, social customs and relationships where smoking is seen as essential in social communications was the most important barrier to the implementation of smoking cessation intervention. At the individual level, migrant-related features such as low education, high mobility and poor integration with local residents, together with poor health beliefs and attitudes added to the challenges of implementing smoking cessation intervention. At the programmatic level, the role of small-team leaders was generally positive, although limited due to their busy work patterns and poor powers of enforcement.

**Conclusion:**

Achieving successful smoking cessation intervention in factories could be challenging with many migrants, as multi-level factors including social context, intervention delivery, individual and migrants’ characteristics play an important role in shaping the implementation of the intervention. Our study suggests the importance of tailoring interventions for the migrant factory workers.

**Trial registration:**

ChiCTR-OPC-17011637 at Chinese Clinical Trial Registry. Retrospectively registered on 12th June 2017.

## Background

Tobacco use is a leading risk factor for almost all non-communicable diseases, causing the deaths of nearly 6 million individuals each year globally. China is the world’s largest producer and consumer of tobacco [1]. With more than 300 million smokers, the prevalence of smoking has most recently been recorded as up to 27.7% (52.9% for male and 2.4% for female) [2]. China has made positive progress in tobacco control since joining the World Health Organization (WHO) Framework Convention on Tobacco Control in 2003 (effective in 2006), despite having the highest smoking rate in the workplace (up to 70%) in the world [3]. In November 2014 the Ministry of Health of China launched a public consultation on potential anti-tobacco regulation in public places, including all indoor public places, workplaces and public transportation facilities [1]. However, to date this regulation has not been officially issued and only a few cities have launched the anti-tobacco regulations in public places.

China has witnessed an unprecedented population mobility following the industrialization, urbanization and economic growth over the last three decades. Many migrants left their rural hometown to seek better livelihoods in better-off cities and the total number of internal migrants had reached 250 million by 2017 [4]. Internal migrants are often marginalized due to the household registration *(‘hukou’)* system that often excludes them from local social welfare. In China, each citizen must be registered at birth, with an urban or rural *Hukou* in their original place of birth. Moving to a new place especially bigger or richer cities does not warrant the change of the *Hukou* status unless it meets the threshold of the local registration policy. This is often challenging for many migrants who tend to have lower education levels and restricts their opportunities for attending free local public schools and enjoying local health insurances. Previous studies have shown that the smoking rates among migrants were up to 46.9–55.3% for men and 1.8–1.9% for women [5, 6], similar to that of the general population (men 52.9%; women 2.4%) [2]. Higher smoking rates among internal migrants could be due to their high working pressure and poor social integration, together with their poor socio-economic status such as lower education and income [5, 7]. To date, tobacco control intervention, along with other risk factors reduction interventions, is rarely reported among the internal migrants, especially those working in factories.

Many studies have explored the factors that influence smoking cessation initiatives among patients with mental health diseases [8–10], patients with other chronic diseases such as hypertension [11, 12], TB patients [13], women [14, 15] and in the hospital settings [16, 17]. These studies have identified a variety of factors associated with the implementation of smoking cessation initiatives, including psychosocial factors (e.g., stress, self-efficacy, motivation and willpower), economic factors (e.g., cost), environmental factors (e.g., social support, peer support and pressure and supervision), risk perception and health awareness, provider awareness and attitude, to name but a few [8–17] .

There have been very few studies, especially qualitative studies that specifically look into the factors influencing smoking cessation interventions in the workplaces, especially in the manufacturing settings with many migrant workers. This study aims to identify aids and barriers to implementing a comprehensive pilot intervention to reduce smoking among migrant workers in factories in China. The details, including the design and effectiveness of the pilot intervention were published in this journal [18].

## Methods

This was a qualitative, process evaluation study embedded in the above-mentioned intervention in Zhongshan city of Guangdong province, a southern province with the largest number of migrant workers in China [18]. Zhongshan city is located in the Pearl River Delta Areas of Guangdong, and is one of the major manufacturing cities. The main component of the intervention was adapted from the WHO recommended ‘5As’ model (‘Ask, Advise, Assess, Assist, and Arrange’) for tobacco control [19]. This was designed into a brief, pragmatic and comprehensible group-counseling package, which included the ‘5As’ smoking cessation guidelines and training modules. This package was delivered by a team leader who had been selected and trained to deliver group counseling to six to eight members and make plans for smoking cessation and follow-up. Based on the group counseling guidelines, the team leaders Asked about their members’ current smoking status, Advised smokers to quit in a clear, strong and personalized manner, Assessed their readiness of quitting, and for those willing to quit, Assisted with individual quitting plans and specialist cessation methods, and Arranged follow-ups in a flexible way. A 5Rs (Relevance, Risks, Rewards, Roadblocks, Repeated) strategy was used to motivate the participants not willing to quit. Supplementary interventions were delivered to support smoking cessation, such as the use of a local social media (WeChat), delivery of open lectures, the distribution of self-help materials (leaflets and booklets) and the display of educational posters (Table 1). Quality control was implemented through collection of the weekly recording forms and field visits to the intervention factories on a two-weekly basis.

**Table 1:** Descriptions of the comprehensive smoking cessation intervention

Main interventions	Supportive interventions	
WHO 5A based group counselling	Wechat “Tobacco-free factory”	Conventional methods
● Development of WHO 5A based group counselling materials including smoking cessation guidelines and training modules; ● Training team leaders; ● Team leaders conducted group counselling for smokers: ✓ Group counselling once per week: 10 to 15 min each time; ✓ 5As (‘Ask, Advise, Assess, Assist, and Arrange’) and 5Rs (Relevance, Risks, Rewards, Roadblocks, Repeated) for participants not willing to quit; ✓ The first follow-up visit within the first week of initiation of smoking cessation and since then once every two weeks.	A multifunctional background interface to deliver tobacco-related text, picture or video messages to help smokers to quit every 2 weeks; A real-time chat room for participants to seek assistance from the team leaders, and share quitting experiences with other participants; Team leaders conducted tobacco-related counseling for participants without WeChat once a week;	● Posters, pamphlets, leaflets, videos; ● Open lectures

Source: [19]

The study employed a controlled before and after design to understand the feasibility of such intervention. The study recruited 149 and 166 participants from the intervention and control arm respectively. In the intervention arm, 91% were male, and 30% were under 36 years old. Nearly 80% were educated at or below secondary school level. Less than 40% had an average family income over RMB3000. Nearly 70% had left their hometown for more than 10 years. Nearly 80% were married and were living with their families, which was where they lived now. The intervention was implemented for 3 months from October to December 2015, significantly improving smoking-related knowledge and attitude in the intervention arm, compared to the control arm, but not the smoking rate [18].

This paper draws on the interview data collected at the three-month follow up of intervention, including twenty semi-structured interviews conducted in the intervention factories. In-depth interviews are a powerful method for generating descriptions and interpretation of people’s social world. A purposive sampling technique was used to select participants, based on their relevance or participation of the project. Interviewees included two managers, eight team leaders and ten migrant workers who had successfully quit or failed to quit smoking in the intervention factories. The interview topics were centred on questions such as perceived effects of intervention, health knowledge and beliefs, social, individual and programmatic/intervention factors which might affect the implementation of the intervention. The interviews were conducted by researchers experienced in qualitative studies in public health. The first author of the paper had PhD training in health systems and social science, especially in the qualitative method. He was responsible for ensuring the quality and consistency of the interviews. In general, the researchers had good interactions with the interviewees, who were encouraged to share their experiences and views of the intervention frankly, although the interviews were sometimes conducted in busy and noisy settings. The factory managers assisted the organization of the interviews, and made appointments and invited interviewees as relevant. Informed consent was obtained from all participants in the study. Each interview lasted approximately 30 min, and was audio-recorded with permission of the interviewees. The recording was transcribed by the interviewers and checked by at least one research team member. We stopped interviewing after conducting twenty interviews, at a stage reaching the ‘saturation point’. Ethical approval was granted by the Ethical Committee of School of Public Health of Sun Yat-sen University in China.

Qualitative data was analyzed using a thematic framework approach, allowing application of an existing framework and inclusion of further emerging themes from the data [20]. The topic guides and transcripts were studied to identify emerging and recurrent themes and a framework table was progressively established and structured. Data analysis, including coding, categorizing and interpretation, with the assistance of Nvivo 10, was mainly conducted by the first author of this paper. The other two researchers (who were co-authors of this paper) revisited the emerging themes and checked through the coding. Consensus was achieved through discussion of any disagreement on the thematic framework and coding. Known as ‘analyst triangulation’, this approach improves the quality and creditability of the research [21].

## Results

Three factors emerged that could influence the implementation of the pilot smoking cessation intervention among migrant workers working in the two Chinese factories: societal, individual and programmatic.

### Societal factors: the influence of social traditions on smoking

Social norms and traditions were the most important barrier for implementing smoking cessation intervention. In many cases, smoking was seen as an essential communication tool and courtesy in social life.

*"There are many people who smoke around me. I think it is a means of social communication and social life. People stay together to share cigarettes with each other, a person who smokes brings many other people to smoke. Such power is so strong.* (Team leader).

*I have tried to stop smoking but did not succeed, because it is sometimes difficult to avoid, especially in the social life. When others pass me over the cigarettes I will generally take a few puffs.* (Migrant worker).

Another team leader also indicated the strong influence from other smokers that hindered the success of smoking cessation.

*For example, in a public place, no one will smoke, and I wouldn’t either. If I see someone smoking around me, I will follow suit. This is a mentality problem- I have attempted to quit smoking for about two or three times, but still fail.* (Team leader).

The interview suggested that small-scale and short-term intervention might not lead to better effect of intervention, since the social customs were deeply rooted. Smoking cessation would require a great range of powerful intervention measures, with societal-level change in social traditions needed to reduce the chances of smoking.

*Smoking may be a social problem; the power of a small team leader is limited. It is best to adopt more deterrent means, so as to bring bigger impact to influence the willpower of smokers.* (Team leader).

*The intervention should not entirely depend on the self-consciousness of the smoker to decide whether to stop smoking or not. We should realize that smoking is not good for the heart and other health problems. The habit, culture and custom of giving cigarettes to others should be changed. During the rural New Year Festival, we used to send cigarettes and wines to our elderly relatives, but it is less so now, so the custom can be changed.* (Team leader).

### Individual-level factors: smoking-related health knowledge, attitudes and behavior and migrants’ characteristics

Our study identified poor health beliefs and attitudes that could add to the challenges of smoking cessation intervention among migrant workers. Some migrant workers even questioned the scientific basis of smoking cessation, and the harm of smoking.

*I know an old man, 70 years old, who has been smoking, but very healthy. He may only have bronchitis without any other diseases. Actually, non-smokers do not necessarily live longer than smokers, and nowadays there is no sufficient scientific evidence, therefore it is difficult to judge the pros and cons of smoking.* (Migrant worker).

Instead, they tried to defend the benefits of smoking such as refreshing the energy.

*But I think smoking is also good, for example, it can stimulate the nerves, and make me refreshing. Especially when someone feels tired, having a cigarette can improve the mental alertness in a short time. It would have illusion when you are so tired and try to do something. Especially when you are driving, having a doze is very dangerous. Therefore, I think smoking may help to relieve the stress.* (Migrant worker).

This interview suggested that migrant workers who had strong desire to quit and took the initiative to do so tended to find it easier to control smoking. When they felt like having a cigarette, they would find snacks or chewing gum.

*I gradually reduced smoking. While working I wanted to smoke, then I bought chewing gum, and other snacks. When I saw others smoke, I just went away.* (Migrant worker).

Our interviews suggested it would be difficult for smokers without strong confidence or determination to quit smoking, as it was easy to relapse.

*I just started to quit, with big determination at the beginning for the sake of health, but now get tired and have given it up.* (Migrant worker).

*In my opinion if you do not have confidence in quitting smoking, you had better not try, because the more times you quit, the higher level of addiction.* (Team leader).

The relatively low socio-economic and educational status of migrant workers could also prevent them from understanding important health education messages.

*Researchers from Sun Yat-sen University came to give us a lecture; we understand some of what they talked about but not the others. We will not understand if the knowledge is too deep.* (Migrant worker).

Despite most of the migrants living with their families, the interview revealed poor integration of migrant workers with the local residents and social life. They were prone to loneliness and boredom, due to poor communication and interactions with the local residents. This loneliness and boredom, reacting with the tobacco addiction, could prompt them to smoke to pass time.

*The reason for smoking is that my usual work is not too busy and quite boring. When seeing others putting the cigarette into mouth, I just want to smoke. At first, I would feel dizzy……. I do know that smoking is not good and harmful for health; this is also indicated on the cigarette packets, but I still want to smoke. It’s too boring….* (Migrant worker).

The mobility of migrant workers added to the challenges of implementing the smoking cessation intervention. During the intervention period, some migrant workers changed jobs, contributing to a high loss to follow-up rate (35%) [18].

### Programmatic factors: the role of small-team leaders in group counseling

The team-leader led group counseling was the main approach for intervention. The regression analysis showed that attending the group counseling sections significantly contributed to stopping smoking or improving smoking-related knowledge and attitudes [18]. The interviewees reflected that education from some team leaders was quite powerful as they shared their own successful experiences of smoking cessation with migrant workers. Some team leaders also set up regulations and punishments within the team. For instance, a penalty of RMB 50 would be applied if the worker was found to smoke, or smoking relapsed, A team leader showed great consciousness of smoking cessation with the interviewer:

*Education is the key to stop smoking. Smoking costs money and is harmful for the health of ourselves and others. I used to smoke heavily, one pack per week. But I was willing to quit and when I want to smoke, I would think of my children as I don’t want to affect their health.* (Team leader).

However, the role of a team leader was reportedly limited. Due to their work, they could only conduct education or remind their teammates to not smoke occasionally, mainly in team meetings or during breaks.

*I examined their smoking status about once every two weeks. When seeing them smoking, I would only provide limited education to them.* (Team leader).

*I talked to all the members in my team face to face during the working hours, but only about three times in two months*. (Team leader).

On the other hand, the interview reflected the limited power of the team leader in providing smoking cessation support:

*Someone would say, what is the relationship between you and me, why do you want to forbid me from smoking? If they do not want to give up smoking, my advice would be useless. After all, it is not you who pay* them the salary. (Team leader).

The interviewees generally appreciated the variety of the intervention components especially the education using video, pictures and social media. Consistent with our published results [18], interviews with the team leaders and members reflected the positive role of modern Chinese social media WeChat in providing educational and warning messages about smoking. However, our interview suggested that the messages delivered by our intervention were difficult to remember and that there was a lack of information on how to deal with smoking relapse. In general it was very difficult for most smokers to stop smoking in a short period of time.

*Most people have this intention to quit smoking, but to quit or not depends on themselves. It is not possible to quit in one or two months, at least we need half a year to know if they have successfully quitted smoking.* (Team leader).

## Discussion

To our knowledge, this is the first study reporting the barriers and enablers in implementing the 5As-based comprehensive smoking cessation intervention among migrant workers in China. Previous studies have examined provider and consumer factors influencing the implementation of the evidence-based “5A’s” in community mental health centers in greater Baltimore [22]; examined cancer patients’ perceptions of 5A’s model implementation by their oncology health care providers [23];examined Chinese pediatricians’ adherence to the 5A’s counseling practices with smoking parents, and identified factors associated with these practices [24]. These studies identified the challenges of implementing the 5A’s based smoking cessation counselling service, such as perceived lack of patient interest in smoking cessation [22], partial implementation of the 5A’s model. Similar to another study [25], this study identifies societal, individual and programmatic-level factors that influence the implementation of the pilot smoking cessation intervention among migrant workers working in two Chinese factories. These results may help to explain no significant improvement in smoking behavior as reported in our quantitative study [18].

Consistent with other studies [26, 27], our study suggests the social origin of smoking and social norms, culture and relations as the most important barriers for implementing smoking cessation intervention among migrant workers. In China, smoking is essential in many social events and giving cigarettes to friends or guests is recognized as courtesy. These traditional values and the role of smoking in the social capital are further worsened by the tobacco industry [1]. Implementing the smoking cessation intervention would be challenging, given the deeply-rooted social norms of smoking as social communication, which the intervention failed to address. While mass education efforts are needed to influence the public’s perception of social behavior, health promotion and intervention on tobacco control should prioritize the change of social norms that may affect smoking-related behavior change at the individual and population level [26].

At the individual level, migrant-related features such as low education, high mobility and poor integration with the local residents, together with poor health beliefs and attitudes added to the challenges of implementing smoking cessation intervention. To some extent, the intervention failed to consider the migrants’ characteristics (e.g., low socioeconomic status) sufficiently as migrant workers reflected that behaviourial change education messages were not easy to understand and remember. Loneliness or isolation, due to poor communication and integration with the local residents might trigger smoking and smoking-relapse. Similar to other studies [28], we found a lack of social support and poor social integration among migrants in China. This study thus suggests the importance of providing psychosocial support and facilitating social integration in achieving smoking cessation among migrant workers. Knowledge and attitude play an important role in the initiation and maintenance of smoking cessation [29]. This study suggests that there is negative impact of poor health knowledge and beliefs on smoking cessation among migrant workers. Despite our quantitative study suggesting positive improvement of smoking-related knowledge and attitude, this study identifies poor knowledge and beliefs that smoking would even lead to ‘better’ health. This problem could be related to the generally lower socioeconomic and educational status of the migrant workers [5, 7]. However, as another study has suggested, smokers with poorer education tend to believe in the benefit of smoking over withdrawal symptoms compared to smokers with better education [30]. Similar to other studies [27, 31, 32], our study suggests the importance of confidence and motivation in quitting smoking. For instance, one study has suggested that smokers with more confidence in quitting tend to be successful. [31]. Smokers who succeed in quitting tend to report high self-efficacy and motivation from post-treatment to follow-up [32]. These merits are particularly important to address smoking relapse, which is often caused by stress, lack of the pleasure previously obtained from smoking, and the smoking environment [27]. Therefore, education and motivation should be given due attention alongside smoking-related knowledge and attitude improvement strategies, in smoking cessation intervention, especially in factory settings with many migrants.

At the programmatic level, the role of small-team leaders was generally positive, although it remained limited due to their busy work pattern and poor enforcing power. The team leader based approach brought the intervention closer to the migrant workers. Other studies have also suggested the importance of peer or social support in smoking cessation [16, 33]. For instance, one study suggested that pressure from a non-smoking spouse helps the smoking partner to cut down [16]. In this study, the perceived effect of education from the team leaders is found to be limited. This could be due to the demands of work and concerns about wage loss in labor-intensive industries. On the other hand, our study suggests the poor authority and influence of team leaders in conducting smoking cessation education. This is particularly true when the team leaders cannot establish themselves as role models for smoking cessation. Given the limitation of the team leaders, future intervention could involve non-smoking migrant workers as ‘coaches’ or ‘helpers’ to their smoking peers. Our study suggests the supplementary role of traditional and modern media in providing education. Popular media such as the WeChat program could provide an innovative intervention platform with opportunities for timely interaction among team leaders and members. There has been a recent emergence of m-health complements strategies towards prevention and control of chronic conditions in low- and middle-income countries [34].

While our study suggests the importance of addressing the barriers to implementing smoking cessation support intervention at the society, individual and programmatic level, the government should speed up the process of enacting and implementing smoke-free polices in the workplaces including the manufactory factories. Studies [35, 36] have shown that implementing smoke-free laws and policies could increase cessation and reduce smoking among workers and the general population.

This study improves the evidence for implementing smoking cessation interventions among vulnerable population in the workplaces, concerning societal, programmatic and individual- level factors. We believe key themes generated from this study are not unique in the study area, and so could be used as references for the smoking cessation and other public health educational interventions in similar contexts, especially in factories. As with other health interventions [37, 38], the ‘floating’ nature of the migrants is a challenge for intervention delivery. We could not trace and interview those smokers who were lost to follow up. The generalizability of this study might be limited as it was a pilot intervention study conducted in the two factories in China. Recall bias and subjectivity embedded within the qualitative study could exist, although efforts such as team triangulation were used to improve the rigor and creditability of the study.

## Conclusion

Achieving successful smoking cessation intervention could be challenging in the manufacturing factories with many migrants, as multi-level factors including social context, intervention delivery, individual and migrants’ characteristics play an important role in shaping the implementation of the intervention. Our study suggests the importance of tailoring the intervention for migrant factory workers. Strengthening the intervention could benefit from optimizing team leader and peer support with accessible and comprehensible messages in traditional and modern educational platforms, providing psychosocial support and prioritizing the addressing of smoking-related social norms, health beliefs and attitudes of the migrant workers.

## Data Availability

The datasets generated and/or analyzed during the current study are not publicly available because of privacy concerns and the qualitative nature of the data. The datasets are available from the corresponding author on reasonable request.

## Abbreviations

5As: Ask, Advise, Assess, Assist and Arrange
5Rs: Relevance, Risks, Rewards, Roadblocks, Repeated
WHO: World Health Organization
